# What’s Special about Human Imitation? A Comparison with Enculturated Apes

**DOI:** 10.3390/bs6030013

**Published:** 2016-07-07

**Authors:** Francys Subiaul

**Affiliations:** 1 Department of Speech & Hearing Science, The George Washington University, 2115 G Street, NW # 204, Washington, DC 20052, USA; subiaul@gwu.edu; Tel.: +1-202-994-7208; 2 Department of Anthropology, Center for the Advanced Study of Human Paleobiology, The George Washington University, 2115 G Street, NW # 204, Washington, DC 20052, USA; 3 GW Institute for Neuroscience, The George Washington University, 2115 G Street, NW # 204, Washington, DC 20052, USA; 4 Mind-Brain Institute, The George Washington University, 2115 G Street, NW # 204, Washington, DC 20052, USA

**Keywords:** imitation, social learning, cognitive evolution, human uniqueness, enculturation, home-reared apes, Do-As-I-Do training, language trained apes, primates, children

## Abstract

What, if anything, is special about human imitation? An evaluation of enculturated apes’ imitation skills, a “best case scenario” of non-human apes’ imitation performance, reveals important similarities and differences between this special population of apes and human children. Candidates for shared imitation mechanisms include the ability to imitate various familiar transitive responses and object–object actions that involve familiar tools. Candidates for uniquely derived imitation mechanisms include: imitating novel transitive actions and novel tool-using responses as well as imitating opaque or intransitive gestures, regardless of familiarity. While the evidence demonstrates that enculturated apes outperform non-enculturated apes and perform more like human children, all apes, regardless of rearing history, generally excel at imitating familiar, over-rehearsed responses and are poor, relative to human children, at imitating novel, opaque or intransitive responses. Given the similarities between the sensory and motor systems of preschool age human children and non-human apes, it is unlikely that differences in sensory input and/or motor-output alone explain the observed discontinuities in imitation performance. The special rearing history of enculturated apes—including imitation-specific training—further diminishes arguments suggesting that differences are experience-dependent. Here, it is argued that such differences are best explained by distinct, specialized mechanisms that have evolved for copying rules and responses in particular content domains. Uniquely derived social and imitation learning mechanisms may represent adaptations for learning novel communicative gestures and complex tool-use. Given our species’ dependence on both language and tools, mechanisms that accelerated learning in these domains are likely to have faced intense selective pressures, starting with the earliest of human ancestors.

## 1. Introduction

Is there anything special about human imitation? Various large-scale studies that have included humans and non-human apes (henceforth, apes) have reported that relative to young human children, apes are poor imitators [1,2]. That is, apes—relative to human children—appear to have difficulties learning and reproducing observed responses. Most comparative psychologists agree that the human imitation faculty has two distinguishing characteristics, its breadth and its high level of accuracy or precision [3,4]. Between eight and 16 months of age, children begin to successfully imitate across many different domains from copying sounds (linguistic and non-linguistic, alike) to transitive and intransitive actions and gestures varying in visual opacity to behavioral and cultural conventions; reproducing all with high fidelity [5,6,7]. In contrast, various authors have noted that great apes, when presented with object-based tasks, for example, are more likely to achieve the same goal using idiosyncratic means, than they are to copy the demonstrated means [8,9,10,11]. Studies reporting imitation in apes have tended to use tasks and/or involved responses that are highly familiar and already present in the subject’s behavioral repertoire [4,12,13,14,15,16]. Evidence of “novel” imitation, whereby apes copy a behavior or response that is new, unfamiliar or untrained, is rather scant [17]. However, various researchers have suggested that apes subjected to various kinds of human-specific rearing or skill training (such as language and imitation)—henceforth, “enculturation”—may represent an important exception. In fact, it has been argued that some of these apes represent “definitive evidence of the capacity for action imitation…” [18] (p. 840).

### 1.1. The Enculturated Mind

Enculturation has various meanings in the comparative literature [19] but, broadly, refers to animals that have been exposed to different levels of human rearing or training. That is, these are animals with species atypical rearing histories. Enculturation ranges from raising animals as “foster children” [20,21,22] to explicit pedagogy in a particular domain like language [23] and social (imitation) learning [18]. This review uses this broad conceptualization of enculturation and focuses on great apes (chimpanzees, bonobos, gorillas, and orangutans) that have been reared by humans, provided with language training, and/or taught to “do as I do.”

A range of ideas have been put forward to explain the (potential) impact of enculturation on the mind—otherwise referred to as the “enculturation hypothesis” (see analyses by Bering et al. [19], Tomasello and Call [24] and Lyn et al. [25]). However, there is disagreement as to the degree and scale of changes affected by enculturating non-human minds [19,24,26]. Arguably, the most extreme version of this hypothesis posits that immersion in a human rearing environment fundamentally transforms ape cognitive development, making it more human-like. As a result, non-human minds that are enculturated develop cognitive skills and abilities that are species atypical and, otherwise, uniquely human (see Bjorklund and Pellegrini [27], Deacon [28], Tomasello and Call [24]). Alternatively, others have argued that enculturation leads to only domain-specific improvements, rather than large-scale cognitive changes (e.g., theory of mind [27,29,30] and communication [25]). Others have suggested that enculturation—or living in a captive environment—depresses, rather than enhances, cognitive development in general and may negatively impact social cognitive development in particular [31,32]. Finally, there are those who have argued that enculturation does not fundamentally alter species-typical cognition at all. These theorists are particularly skeptical of the possibility that enculturation can result in the development of new species atypical or uniquely human cognitive skills [19,26,33]. According to Vonk and Povinelli [26] (p. 344), enculturated apes learn “a greater breadth of skill sets for coping with human social and material culture”. That is, enculturation does not fundamentally change ape cognition per se, but instead shapes apes’ species-typical behavioral responses to suit this species *atypical* rearing environment.

In order to evaluate whether humans possess uniquely derived imitation skills and whether such skills are the product of unique rearing (and learning) experiences, this review focuses on the imitation performance of enculturated apes, arguably, the best ape imitators [18,29]. Specifically, how the performance of enculturated apes is like or unlike that of young preschool age children and non-enculturated apes in controlled experimental settings. (This review excludes field studies because while important for the purposes of characterizing species-typical behaviors, they lack the experimental control necessary to isolate domain-specific imitation learning [34]). If enculturated apes can learn to imitate and their performance is no different from that of preschool humans, then differences between human and great ape imitation are not due to specialized cognitive machinery for imitation or social learning, but to missing social-cultural input as suggested by Tomasello and colleagues [29,35] and more recently by Heyes [36,37,38]. 

In particular, this review looks critically at imitation performance in various domains believed to tax different cognitive and representational skills [6,39,40,41]. This approach differs from most comparative studies, which have made strict categorical distinctions between imitation—defined as learning and faithfully reproducing a demonstrated response [42]—and other forms of social learning—defined broadly as any socially-biased learning [12,43]. In that view, social learning—in addition to imitation—also includes: emulation [9,10,11], whereby an individual copies the end-result [44], object movements [45] or intention [16] of the model’s actions without copying the means demonstrated [46]; Stimulus/local enhancement [44,47], where an observer’s attention is directed to a particular object(s) or location(s) but learning results from happenstance or trial-and-error learning; and response facilitation or priming [12,48,49,50,51], where previously learned sensory-motor schemas are activated resulting in the associated motor response being released in the presence of the corresponding visual stimuli [52]. This phenomenon has also been referred to as automatic imitation [53] and familiar imitation [40,46,54,55]. (For a more complete review of all social learning terms see Zentall [50]). In contrast to these earlier analyses, the present review assumes that imitation is not mediated by a single, unitary mechanism operating at the exclusion of other forms of social learning [56]. Rather, the framework used here assumes that imitation is mosaic and as such there are multiple, specialized mechanisms mediating different forms of social and imitation learning in particular tasks and content domains [40,46,57]. These different mechanisms may act independently or in concert with one another depending on the experimental context. For an alternative mosaic model of imitation see Bates and Byrne [58]. Subiaul’s mosaic model [40,46,54], in particular, proposes that in some tasks, imitation is mediated by domain-general mechanisms alone (e.g., executive functions, associative learning or priming [36]), but in others, more specialized mechanisms are necessary (e.g., simulation [59], hierarchical goal organization [48], and copying item- versus motor/spatial-specific responses [40]). As such, the present review aims to assess whether the imitation of particular content/information types, representing specific imitation domains, differentiates human imitation skills from those of enculturated apes. In doing so, this analysis seeks to shed light on whether imitation in particular content domains are a unique feature of human cognition or a product of social-cultural (learning) experiences.

### 1.2. Assessing Domain-Specific Imitation Performance

Imitation tasks may be differentiated by their opacity, transitivity and novelty [6,17,58,60]. Research from the developmental, computational and brain sciences has suggested that these task features present unique computational demands. Consequently, imitation in these tasks is associated with activation in distinct cognitive and neural systems [6,54,60,61,62]. For instance, actions varying in visual opacity range from opaque—where there is no visual access when performing a target action (e.g., oral-facial gesture)—to transparent—where there is direct visual access when performing a target action (e.g., hand clapping). Actions varying in transitivity range from transitive actions directed toward an object with an end-state/goal (e.g., hammering a nail) to intransitive actions or gestures that do not involve an object (e.g., waving goodbye). Finally, these different types of responses could be novel to the subject, requiring the online development of a sensory-motor representation, or they may be familiar to the subject, requiring subjects to recognize and accurately recall from their existing motor repertoire the corresponding response.

Distinguishing what is novel and what is familiar in imitation research is a complicated undertaking. Such an undertaking is made harder by the fact that behaviors often include both novel and familiar elements [63]. Research from the cognitive neurosciences suggests that the execution of novel and familiar elements in imitation tasks is likely mediated by associative learning processes [64,65,66,67], semantic and working memory systems [39,61,68,69], and cognitive simulation mechanisms [70,71,72,73]. Here, we suggest that “novelty” should be evaluated along three dimensions: responses, objects and (experimental) contexts. Below each is operationalized in turn. It is important to note that responses, objects, and contexts are features of a given task. This is a proposed framework by which one might operationalize “familiarity” and “novelty” for each of these features. These descriptions are not (and do not correspond with) imitation or social learning terms.

Responses are novel in so far as they are: (a) arbitrary or meaningless (i.e., not connected to a specific—semantic—memory representation); or (b) unfamiliar and do not already exist in the behavioral or cognitive repertoire of an individual; and (c) have not been previously reinforced. This operationalization of response novelty corresponds with Heyes’ [74] concept of elemental novelty. Responses are also novel if, several familiar (or meaningful) responses, species-typical behaviors or previously reinforced actions are executed in a new, hierarchically organized [48,63] sequence. This last operationalization corresponds with Heyes’ [74] concept of sequential novelty. For example, putting one’s palm at the top of one’s head or touching one’s forehead with one’s index finger (familiar) versus putting one’s palm at the top of one’s head immediately followed by touching one’s forehead with one’s index finger (sequential novelty) or putting one’s thumb with the rest of the fingers in a fist on top of one’s head (elemental novelty). Note that this last behavior, though absent from most individual’s behavioral repertoire (i.e., it is a meaningless, rare or never-observed gesture) is, nonetheless, well within a healthy individual’s behavioral and motor capabilities.

An object’s novelty should be judged by its affordances and familiarity to the subject. Thus, an object is novel if its elements have one or more of the following features: (a) unfamiliar to the subject (i.e., the subject has not interacted with the same object or similar objects with similar affordances in the past); (b) functional elements are causally opaque and/or arbitrary; or (c) ecologically meaningless, involve objects or tools that are not part of the subject’s natural—or experiential—repertoire, such as the use of sticks or rods, which great apes habitually (and spontaneously) use to retrieve rewards in captivity as well as in the wild [4,75,76].

Context novelty—specifically, experimental context—is novel when: (a) tasks and experimental procedures involve novel actions, responses and/or novel objects (defined above); (b) responses are not causally yoked to previous responses, where one response necessitates the other (i.e., task lack “enabling relations” [77,78,79]); and (c) specific responses or task features have not been previously reinforced. For instance, Subiaul and colleagues, tested rhesus monkeys [80], orangutans [81,82], and preschool age human children [46,54,55,83,84] on a computerized serial learning task or “cognitive task” [85] that requires participants to touch individual picture items on a touchscreen—corresponding to distinct objects—in a specific order. From trial to trial, objects change spatial location, preventing subjects from learning a specific motor or spatial response. In this task, individual responses are familiar (touching and pointing to an object on the screen). However, the task context is, nonetheless, novel because: (a) familiar responses are directed to several new objects; (b) responses are causally arbitrary; and (c) specific responses to particular objects have not been previously reinforced.

The meaning of “context” used here differs from that of Henrich and McElreath [86]. In that view, context includes features of the model as well as the frequency of a given modeled behavior. It also differs, somewhat, from the meaning of context used by Bates and Byrne [58] (i.e., contextual imitation), where familiar actions are directed toward new objects, for example. That is, the new object or task is the new context. Here, a new experimental context includes new objects, new actions or both.

In order to determine “novelty,” most studies use participants’ performance in a Baseline condition, where participants are observed for a set period of time at play on their own or while interacting with “virgin” objects; that is, objects that are new or unfamiliar to the subject [87]. Performance on these conditions is then used as a baseline rate of learning. Such conditions are undoubtedly useful and should be included whenever possible. Nonetheless, we must bear in mind that a Baseline observation period lasting several minutes—even one that extends for hours or days—almost certainly under-estimates the full range of responses present in participants’ behavioral and experiential repertoire [63]. In other words, Baseline conditions almost certainly under-estimate subjects’ knowledge and behavioral repertoire.

In this analysis, we will highlight different types of imitation skills that are likely to be shared and uniquely derived among the great apes. Below, we use these criteria to review several studies that assess imitation learning in enculturated apes [4,13,18]. Table 1 and Table 2 summarizes apes’ performance across the different content domains, focusing on novel actions (Table 1) and intransitive and bi-manual actions (Table 2).

## 2. Candidates for Shared Social and Imitation Learning Mechanisms

### 2.1. Imitation of Familiar Transitive Actions on Objects

These object-directed responses correspond to imitating familiar, single actions on an object that is external to the body. Transitive actions may be further divided into transparent or opaque. An example of a familiar transitive action on an object that is transparent and can by guided by vision is kicking a ball. An example of a familiar opaque transitive action, which cannot be guided by vision, would be hitting a drum overhead. 

Various studies have demonstrated that enculturated apes [22,94,95,96,97,98] and to a lesser degree, mother-reared or peer-reared apes without any enculturation experience (henceforth, “non-enculturated” apes) [47], can successfully imitate familiar, transparent transitive actions on objects. In one study, Tomasello and colleagues [35] compared two groups of apes, enculturated and non-enculturated chimpanzees and bonobos with two groups of human children: 18- and 30-month olds. Results revealed that when copying a single familiar action—present in the behavioral repertoire of all research participants—such as spinning, twirling or standing a variety of objects, both enculturated apes and 30-month-old children successfully imitated a significant number of actions. However, 30-month-old toddlers were almost twice as accurate as the enculturated apes. Non-enculturated apes failed to imitate most actions. There were two actions where the enculturated apes out-performed children, including brushing foam on the floor and wiping foam using a squeegee. One explanation may be that these two tasks may have been more familiar to (or inadvertently reinforced in) the enculturated group in comparison to the other experimental groups, explaining their high levels of accuracy in these tasks.

Together, these studies demonstrate that apes—enculturated and non-enculturated alike—can learn to copy relatively simple transitive actions on objects. General processes, rather than specialized mechanisms, likely mediate this type of familiar imitation [38,74]. An open question is whether there are species differences in the copying of familiar but opaque transitive actions relative to the copying of familiar but transparent transitive actions. Future research should also explore if any species differences or specific difficulties copying opaque actions that are familiar to the subject can by explained by motor and sensory differences or differences in representational mechanisms involved in either the simulation or translation of observed actions into a matching response.

### 2.2. Imitation of Familiar Transitive Actions on the Body

These responses involve single, familiar actions that are directed toward the body such as touching one’s leg or face. As above, transitive actions may also be transparent or opaque. Custance and colleagues [18] report on the performance of two chimpanzees, KA and SC, who were peer-reared until the age of four, but were described as having “regular interactions with humans” and, importantly, received 2–3 imitation-specific training sessions, lasting 30–40 min each, five days a week, for several months. Custance and colleagues’ analysis of the data showed that two independent raters agreed that subjects successfully executed nearly half of all demonstrated responses. An analysis of opaque and transparent transitive actions on the body showed that subjects imitated both types of actions at equivalent rates. Call [92] replicated both the procedures and the results reported for chimpanzees by Custance et al. [18] with a language trained orangutan, Chantek. Chantek’s performance was overall better than those reported for the other two chimpanzees, KA and SC. His level of accuracy for both opaque and transparent transitive actions on the body ranged from 67% to 100%. Tomasello and colleagues [35] reported similar results for two of three opaque transitive actions on the body, placing an object on the head and draping something around the neck. For each of these actions, two of the three enculturated bonobos imitated the demonstrated actions perfectly. Tomasello and colleagues also tested three non-enculturated chimpanzees and a group of 30-month-old human toddlers. Whereas none of the non-enculturated chimpanzees imitated the two opaque transitive actions on the body, more than half of the 30-month-olds copied these actions with high fidelity. The performance of the 30-month olds was comparable to what was observed for two of the three enculturated bonobos and chimpanzee.

In sum, while some enculturated apes consistently imitated familiar transitive actions on the body that were opaque (e.g., Orangutan: Chantek; Bonobo: Panbanisha), others copied similar actions but with lower levels of fidelity (e.g., Chimpanzee: KA and SC). These results suggest that all apes may be capable of learning to imitate familiar opaque actions directed toward the body, yet have some difficulties in comparison to humans.

### 2.3. Imitation of Familiar Object-Object Actions and Tool-Use

This class of actions involves putting two familiar objects in contact with one another. Some of these actions include placing one object inside or on top of another object, for example. Other object–object actions are best described as tool use, where a tool is used to affect the operations of another object. There are various examples indicating that when apes are given familiar tools such as sticks to use with objects, they generally imitate the model successfully. This is true for both enculturated [22,95,96,99] and non-enculturated apes [100,101,102]. Hopper and colleagues, for instance, showed that non-enculturated apes were better at imitating a familiar response (e.g., poke), using a familiar tool (i.e., sticks or rods) in a novel context (food-dispensing machine) rather than a more novel response such as using the rod to lift a lever in the same novel context. Carrasco and colleagues [94] similarly demonstrated that, Lili, an enculturated chimpanzee, imitated with high accuracy, familiar object–object actions such as threading a rope through a buoy handle as well as actions with tools, such as holding a buoy by handles and hitting a barrel. This example demonstrates that enculturated chimpanzees—and, perhaps, all apes—given enough experience executing the requisite responses, can successfully imitate even multi-step sequential actions. The work by Whiten and colleagues suggests that the same is true for non-enculturated apes, even when such familiar actions are directed to novel objects presented in a familiar—ecologically meaningful—context [101,103,104] or “contextual imitation” [58].

### 2.4. “Rational” Imitation or “Goal Emulation”

When observing the actions of others, different classes of representations may support social learning and imitation including, unobservable psychological states such as intentions, unobservable causal states such as gravity and support, observable motor responses, and observable end-states, effects or results [51]. In the case of rational imitation, observers’ responses appear to be guided primarily by unobservable representations—intentions, causes or both—rather than by observable events alone. In a now classic study, Gergely, Bekkering, and Kiraly [105] demonstrated that 14-month old infants, when observing an adult execute an action, consider the goals of the actor’s actions and how such actions are causally (or rationally) constrained. So, for example, when infants saw a model place her hands on a table and then bend over to turn on a light panel using her forehead, infants imitated the model exactly. However, when a different group of children observed the model cover their entire upper body (including their hands) with a cloth, pretending to be cold, and then bent over to turn on the light panel using her forehead, infants did not imitate her action. Instead, they used their hands to turn on the light panel. This result has been interpreted to mean that infants as young as 14 months of age imitate others rationally and flexibly, using an abstract (unobservable) intentional and causal framework.

Buttelmann and colleagues [98] replicated the general procedures used by Gergely et al. using similar familiar responses in a novel experimental context. Enculturated chimpanzees were tasked with activating a novel apparatus using the following responses: sitting on it, kicking it and pressing their head against it. Note that some of these actions were transparent (kick) and others were opaque (head). These actions also varied from familiar (sitting) to novel (head-pressing). Overall, the enculturated chimpanzees were more likely to imitate the model in the hands-free condition, where the experimenter’s hands were free to execute the demonstrated action (mean = 41.7%), than in the hands-occupied condition, where the experimenter’s hands held an object and could not be used to execute the target action (mean = 16.7%). However, in comparison to the imitation of sit (50%) and foot (43.8%) actions, chimpanzees were relatively poor when imitating the novel and opaque head action (18.8%) in the hands-free condition. In addition, this overall pattern of results was only marginally significant on the first trial (*p* = 0.06) and disappeared altogether on the second trial. Given that first trial accuracy results were marginal and the overall effect sizes were small, this study warrants further replication with other enculturated apes. Similar procedures used with non-enculturated chimpanzees, bonobos, gorillas and orangutans yielded almost uniformly negative results [106]. Though, Buttelmann et al. [106] provide some evidence that non-enculturated orangutans may have evidenced greater rational imitation than the other great apes species. Exactly what accounts for this species difference remains unclear and merits further study.

## 3. Candidates for Uniquely Derived Social and Imitation Learning Mechanisms

The evidence reviewed above demonstrates that enculturated great apes and in some cases non-enculturated apes, share some social and imitation learning mechanisms with human children. These include the ability to imitate single, familiar transitive actions on objects and on the body, as well as tool-use involving familiar or meaningful responses. There is also limited evidence that suggests that enculturated chimpanzees but, generally, not the non-enculturated chimpanzees along with other non-enculturated apes (with the possible exception of orangutans) can imitate the use of tools “rationally”. However, enculturated chimpanzees in Buttelmann and colleagues’ [98] study showed specific difficulty copying opaque actions relative to transparent actions. Myowa-Yamakoshi and Matsuzawa [91] similarly reported that five chimpanzees who were nursery-reared and had extensive human contact and training, failed to copy all familiar opaque transitive actions with objects. However, such difficulties were not present in all of the apes tested (e.g., Chantek, KA, SC). Besides a possible domain-specific difficulty copying opaque responses, enculturated apes also show several limitations imitating familiar and novel intransitive actions and novel motor responses with objects, including tools.

### 3.1. Imitation of Intransitive Actions or Gestures

These responses include gestures or motor responses that are not directed toward an object or with a specific function such as grasping. Intransitive actions or gestures include a variety of responses whose underlying neurobiology and cognitive processes may be dissociable. Some distinctions include the familiarity (or “meaningfulness”) of actions [69] and whether responses include bi- or uni-manual actions [107]. In human infants, some have argued that the imitation of opaque responses rely on an internal proprioceptive map and/or an imitation-specific body representation that is amodal [108]. Alternatively, some have suggested that the imitation of opaque gestures (or any other action) is mediated by general processes (e.g., associative learning [109] or priming [48]). However, such general mechanisms cannot explain the imitation of novel gestures on the first trial because novel gestures, by definition, are not present in the individuals’ behavioral repertoire and lack reinforcement history.

Hayes and Hayes [22] reported that several intransitive responses were difficult for Viki—a home-reared enculturated chimpanzee—to execute. They explain that this failure was not because of a failure of attention or motivation but rather, “seemed to involve the execution of the act…Blinking the eyes [for example] seemed to be absent from her voluntary motor repertory…” [22] (p. 452). They go on to note that “[Viki] occasionally did poorly on tasks, which did not appear to present motor difficulties” [22] (p. 452). Custance and colleagues [18], who replicated the procedures used by Hayes and Hayes [22] reported that their two chimpanzees KA and SC failed to imitate certain familiar oral-facial gestures such as tongue and lip protrusions. Washoe, a home-reared (cross-fostered) sign-language trained chimpanzee, also evidenced specific difficulties imitating intransitive gestures, specifically, ASL signs. Regarding the learning of novel ASL signs, Gardner and Gardner [88] (p. 672) concluded that “…very few, if any, of her early signs were introduced by immediate imitation”. They also note, however, that Washoe had difficulty imitating intransitive gestures already present in her motor repertoire, “It was not until the 16th month of the project that we achieved any degree of control over Washoe’s imitation of gestures” [88] (p. 165). Sanders [89], after analyzing 15 video-taped interactions between Nim [110]—a home-reared sign-language trained chimpanzee—and his handlers, similarly concluded that Nim failed to learn new signs by imitation.

However, Carrasco et al. [94] reported that the enculturated chimpanzee, Lili, imitated three different opaque intransitive gestures, including making a U-shaped tongue protrusion on the first trial. Call [92] reported that Chantek’s—a sign-language trained orangutan—rate of imitating oral-facial actions was moderate to high (60%). However, Call reported two significant types of errors. First, while Chantek accurately matched 90% of gross body movements involving whole body parts (e.g., head, arms, body, and legs), he had difficulty imitating specific body parts (34%), such as a finger movement. Second, Chantek was better at imitating actions that involve some form of contact between the hand and the body (70%), than those without (29%). Call notes that this result has been described for most great apes (chimpanzees, orangutans, and gorillas) participating in language acquisition and imitation experiments [95,111,112,113].

Byrne and Tanner [63] offer an excellent case study and analysis of an ape’s ability to imitate intransitive gestures. These authors presented Zura, a zoo-housed Western Low-Land Gorilla, with seven gestures that they believed to be low frequency, not species-typical and otherwise absent from Zura’s behavioral repertoire. Results showed that Zura learned and faithfully imitated four of seven gestures (57% accuracy). However, an in-depth analysis of Zura’s responses before and after the demonstration period revealed that Zura generated one of these gestures *before* the demonstration. The three remaining gestures were detected in an analysis of five and half years worth of video cataloging Zura’s gestural repertoire.

These results show that while great apes appear unable to copy gestures that are meaningless or novel, many show similar difficulties reliably and consistently copying intransitive gestures that are meaningful and familiar to them. However, there is one possible exception: the imitation of intransitive—oral-facial—gestures in infancy. To date, various researchers [91,114,115,116] have demonstrated that two non-human primate species (chimpanzee, rhesus monkeys) can copy opaque intransitive responses soon after birth. For instance, Myowa-Yamakoshi and colleagues [91], using procedures similar to those employed with human infants [117,118], assessed newborn chimpanzees’ ability to copy three target oral-facial gestures: tongue protrusions, mouth openings and lip protrusions. Results showed that infant chimpanzees—during a 20 s response period—generated two of the three target gestures (tongue protrusions and mouth openings) more often than any of the alternative oral-facial gestures. The presence of neonatal imitation in human and chimpanzee infants, only if a single gesture is imitated reliably (e.g., tongue protrusion [74,109]), suggests that there are specialized mechanisms for copying at least some opaque intransitive gestures. While some have questioned the veracity of neonatal imitation [74,109], reports that have failed to find evidence of neonatal imitation are often characterized by small sample sizes [119] or short response windows coupled with a large number of demonstrated responses [120]. These last two methodological features, in particular, are problematic because a large number of demonstrated responses, presented during a short response window to subjects characterized by poor executive functioning skills [121,122], increases the likelihood of interference effects, masking or dampening the expression of neonatal imitation.

In sum, despite rhesus macaques’ and chimpanzees’ ability to copy oral-facial gestures during infancy [91,114,115,116], the evidence reviewed above indicates that adult chimpanzees (along with the other great apes) have a specific difficulty doing the same. These results are in contrast with those reported for typically-developing children who by 18 months of age perform at ceiling on tasks involving similar intransitive gestures [7,41,123]. Nonetheless, the discrepancy between neonatal and adult imitation, is consistent with the hypothesis that there are distinct mechanisms or cognitive processes supporting oral-facial imitation in infants and adults [124], the development of which seem to follow divergent paths in humans and great apes. Results are summarized in Table 1.

A fruitful avenue for future research might be to analyze the types of errors made by great apes and humans alike when copying intransitive gestures (as was done by Call [92]). Might the errors be explained by fine motor difficulties, suggesting species differences in peripheral motor and sensory systems? Or, are the differences best explained by motor *planning* difficulties, suggesting species differences in motor simulation mechanisms or bodily representations—a cognitive difference rather than a motor or sensory difference?

### 3.2. Imitation of Novel Transitive Actions on Objects

Virtually all researchers who have presented enculturated apes with novel transitive actions on objects have reported either mixed or negative results. For example, Tomasello and colleagues [35] reported that on a variety of transitive actions that included simple as well as complex (serial) responses with a mix of familiar and novel elements, moderate to low levels of imitation were observed in three language trained apes. Call and Tomasello [90] showed that despite Chantek’s relative success imitating transitive actions directed toward the body [92], he failed to imitate novel transitive actions directed toward an object. Myowa-Yamakoshi and Matsuzawa [95] reported that the five chimpanzees tested reproduced less than 5% of novel transparent transitive responses on objects on the very first trial. This pattern of performance was generally true for both transparent and opaque responses. However, as previously noted, it may be worse for opaque responses. Carrasco et al. [94] reported the opposite pattern, however. For example, Lili performed worse on transparent (mean = 38%) than on opaque transitive actions (mean = 60%). However, some of these actions involved a single response and had various familiar elements such as putting an object on the head, which may have contributed to the differences in results between opaque and transparent transitive actions. In sum, enculturated apes appear unable to reliably and faithfully copy novel transitive actions on objects (cf., Table 2).

### 3.3. Imitation of Novel Object-Object Actions or Tool-Use

As with transitive actions on objects, apes also show difficulties imitating actions involving multiple objects in novel contexts, as well as those involving novel responses. For example, Tomasello et al. [35] reported that three enculturated apes failed to imitate how to use a “grabber pincer” to pick up a piece of cloth that was out of reach. These same individuals also had difficulties with object–object actions that involved bi-manual actions such as reeling in an object. Buttelmann et al. [106] reported similar difficulties among non-enculturated chimpanzees, orangutans, gorillas and bonobos. All of these great apes evidenced specific difficulties copying bimanual actions on objects. In another study, Tomasello and Carpenter [93] demonstrated that three human-reared chimpanzees—Annet, Alex, and Alexandra—failed to imitate most object–object actions such as putting a ring on a latch. Out of four different tasks, one chimpanzee, Annet, failed to imitate any target action. Alex imitated only one of the four target actions and Alexandra imitated three of the four target actions. Curiously, these apes performed better in experimental conditions that included demonstrations of either failed attempts or accidental actions, a result that has also been found in children [54,55,125]. Carrasco et al. [94] reported that Lili did not respond when asked to imitate most opaque object–object actions. When she did respond, she imitated accurately. She did attempt to imitate transparent object–object actions. However, only half of these actions were imitated correctly on the first trial. Lili did not fail to imitate these actions because she lacked the requisite motor skills or motivation. For instance, she failed to imitate putting a straw inside a bowl and covering the bowl with a lid. She also failed to imitate putting stones in a container and shaking it with both hands. These are, perhaps, novel action sequences, but they are actions that are clearly within her motor and behavioral repertoire. Her failure to imitate these basic action sequences is striking given her success imitating familiar, well-trained actions, which she performed at ceiling. These results show that despite being able to copy familiar object–object actions and tools, enculturated apes have a specific difficulty reliably and faithfully copying novel object–object actions and tool-use, as well as bi-manual actions with objects (cf., Table 2).

## 4. Discussion

*Imitation in Enculturated Apes is Different from Imitation in Humans.* Whether non-human primates imitate has been debated for more than a century [126,127]. Yet, there is still much uncertainty as to the uniqueness of our own species’ imitative abilities. Much of that uncertainty centers on how social and imitation learning observed in other animals may be like or unlike those present in humans. A particularly interesting case is that of enculturated apes, which have been reared by humans, provided with linguistic and/or imitation-specific training. Some have argued that (at least some) enculturated apes, in contrast to non-enculturated apes, imitate much like 2.5 year olds [13,29,99]. However, if we are to understand what (if anything) is unique about human imitation, we cannot simply ask, *Can* these apes imitate? Instead, we must ask, *What* can these apes imitate? *How* are these apes imitating? And, do answers to these two questions differ between humans and apes? Otherwise, we run the risk of confusing behavioral outcomes with underlying mechanisms; Falling into the trap of the argument by analogy [128]. When questions are framed in this narrow way, it becomes clear that even enculturated apes have significant difficulty (or are incapable of) copying certain actions in particular domains that are well within their capabilities. Specifically, while enculturated apes generally outperform non-enculturated apes on many different imitation tasks (e.g., [35]), both enculturated and non-enculturated apes alike are more likely to imitate familiar responses in familiar contexts than novel responses in novel contexts. The challenges persist even when the novel responses are composed of familiar actions (i.e., sequential novelty) [22,94]. Adult great apes may also have a particular difficulty imitating intransitive actions, regardless of opacity or familiarity.

Such conclusion may be surprising to some given that enculturated great apes have been thought by many to engage in “true imitation” [4,13,18]. That is, the ability to copy a novel act [42]. Consider the enculturated chimpanzee, Viki. This enculturated chimpanzee is often held up as providing “definitive evidence of the capacity for action imitation in chimpanzees” [18] (p. 180). Yet, according to Hayes and Hayes [22] (p. 452), Viki “…imitated immediately, without preliminary tutoring—provided that she had previously done…[the demonstrated behaviors] in other situations”. Nearly fifty years later, Myowa-Yamakoshi and Matsuzawa [129] (p. 131) came to a similar conclusion when testing novel motor imitation in five enculturated chimpanzees, saying, “actions involving motor patterns that subjects had already possessed were easier for chimpanzees to perform than unfamiliar actions”. Gillespie-Lynch [130] (p. 3) et al. in describing the language training of Washoe [88,131] surmise that “…immediate imitation was more effective for shaping signs than for introducing novel signs…Indeed, Washoe’s early signs were often shaped from spontaneous behaviors…”. However, the problem is not just limited to copying novel responses. Enculturated apes also have problems copying some familiar responses reliably and with high fidelity. Recall that KA and SC [18] as well as Viki failed to imitate familiar oral-facial gestures such as tongue and lip protrusions and Chantek, while imitating transitive actions directed toward the body at near ceiling levels, had significant difficulty imitating similar instransitive actions that were not directed to particular body parts.

Apes’ broad difficulty copying intransitive actions, as well as a specific difficulty copying novel transitive actions with objects are surprising given that in contrast to non-enculturated apes, but like human children, most of these apes had rich rearing histories: Viki was reared as a foster child and taught to “do-as-I-do.” Others such as KA, SC, Lili and Zura had either imitation-specific training or rich social experiences. The difficulties (and general failure) of most of these apes to copy novel transitive actions and various types of intransitive gestures stands in contrast to that of human children who can reliably imitate (often at ceiling levels) in these domains between 12 and 18 months of age [5,7,132]. This review makes clear that apes, regardless of rearing history, have domain-specific imitation deficits. What is unclear—and remains contested—is why? What exactly is the source of such differences? Below, some possibilities for these differences are explored, along with some suggestions for future research and analysis.

### 4.1. Associative and “General Process” Accounts of Continuities and Discontinuities

Heyes [36,52] has offered a framework by which to understand both continuities and discontinuities in social learning across species. In her model, underlying all imitation learning is associative learning, and differences between species are best explained by differences in peripheral sensory and/or motor systems than by central cognitive differences. This theory has some appeal. According to this theory, enculturated apes are better imitators than non-enculturated apes because they have been reinforced to attend to more information (sensory input) from their environment. The more access to information that you have, including reinforcement for matching responses, the better the imitation performance. However, Heyes’ associative model has the following limitations when explaining the pattern of imitation performance reported here:

First, *success in one imitation domain did not lead to success in* other *imitation domains.* Heyes’ ASL model assumes that imitation—regardless of task or domain—is mediated by the same general cognitive processes (e.g., executive functions and associative learning [36,38,52,109]). Because imitation learning relies on the same “core” processes, then success in one imitation task should predict success in other imitation tasks. This should be especially true within task domains, where motor and sensory responses are matched. However, as reviewed above, both enculturated and non-enculturated apes alike succeed in copying various *familiar* transitive actions but, generally, failed to imitate *novel* transitive actions that are motorically similar. Likewise apes’ success copying transitive actions on the body appears to be disconnected from the ability to copy intransitive actions involving the same body parts.

Second, *individual learning within a domain does not correspond with imitation learning in the* same *domain.* Heyes’ framework also predicts that “social and asocial [individual] learning covary” [38]. Various studies with preschool age children have directly tested this claim. Those studies have failed to find any significant association between learning a rule by imitation and learning the same rule by trial-and-error [46,54,55]. There are, presently, no studies with great apes directly comparing covariances between individual learning and social learning within tasks. However, there is a significant body of research showing that chimpanzees [133] and orangutans [134] naturally use tools in the wild to access food resources. While some have questioned whether apes understand the physics underlying such tool-use [33], these same studies have shown that chimpanzees (and perhaps, all apes) can individually learn to use complex tools. Yet, as this review shows, apes have significant difficulty copying novel transitive actions with tools. In short, apes can learn to use tools by individual learning, but they seem to have difficulty (or are incapable of) learning to use new tools by imitation learning. If social and asocial learning were mediated by the same core processes, the fact that apes can learn to use tools by individual learning should predict their ability to use similar tools by imitation. However, that does not appear to be the case.

Another consideration is whether species’ differences in working memory (WM) [135]—the ability to maintain and update information—may explain apes’ poor imitation performance. WM is of particular interest because it has been directly linked to the imitation of novel (but not familiar) intransitive gestures in human adults [39,61,68,69,136]. It is possible that great apes may have poor executive—WM—skills necessary to flexibly manipulate novel information, while inhibiting prepotent (familiar) responses. Certainly, in children, WM constrains imitation, as one would expect [83,137]. However, evidence suggests that chimpanzees have excellent WM, one that is at least at par with that of adult college students [138]. These results are inconsistent with the idea that deficiencies in WM are responsible for apes’ difficulties copying novel responses in different domains.

Finally, *despite imitation-specific training, domain-specific imitation deficits persist.* Heyes’ framework assumes that individuals “learn to imitate” [74]. Consequently, imitation-relevant experience should covary with imitation performance, particularly if imitation performance is mediated by general (non-specialized) processes. Consistent with this hypothesis are results showing that enculturated apes—with significant imitation-relevant experience—generally, outperform non-enculturated apes—who lack comparable imitation-relevant experiences—on gross measures of imitation learning (i.e., aggregating results across tasks and domains) [35,139]. Inconsistent with this hypothesis is the fact that both enculturated and non-enculturated apes—despite their significant differences in imitation-relevant training—show the same types of imitation-specific deficits in the same content domains. For example, both enculturated and non-enculturated apes have difficulties copying intransitive actions and novel actions on objects [4,13].

Heyes [36,38,74] has sought to explain such discontinuities by suggesting that the problem lies in differences in peripheral sensory and motor systems (i.e., “input” or “output” processes). However, such an explanation is unsatisfactory in this case because first, the motor and sensory skills of enculturated and non-enculturated apes are not different. Second, the motor and sensory skills of apes, in general, while likely differing from that of adult humans, have been shown to overlap significantly with those of 2.5-year olds [140]. Specifically, basic sensory-motor skills including *In*-relations (i.e., placing an object(s) in a container of various sizes) and *On*-relations (i.e., placing objects(s) on top of one another) develop at approximately the same time in humans and chimpanzees, while *Next-to* relations (i.e., placing objects adjacent to one another) are present, but underdeveloped in chimpanzees relative to human children [140]. Poti and Parenti [140] (p. 11) conclude that observed species differences “…point to chimpanzees’ relative difficulties in simultaneously considering and coordinating independent positions in space”, indicative of a cognitive representational deficit rather than a sensory-motor (input or out processing) deficit. Finally, chimpanzees’ ability to copy some oral-facial gestures as infants (but less so as adults), further demonstrates that chimpanzees—and, perhaps, all apes—have the necessary motor and sensory competence to imitate bodily actions, certainly familiar intransitive actions, unless, of course, we want to argue that the sensory and motor skills of chimpanzees—including those that have been enculturated—worsen rather than improve with age and experience (a consideration that is unlikely, for reasons noted below in Section 4.3). Instead, such dissimilarities between species’ imitation performance in particular content domains suggest that differences are not due to the quality of the sensory input or motor output but, as Poti and Parenti [140] argue, how sensory input and motor output is processed and represented in the minds of apes and human children.

In sum, Heyes’ associative framework is better at predicting the imitation of familiar actions that are already present in an individual’s behavioral repertoire than at explaining the imitation of novel—unfamiliar, unrehearsed or unreinforced—actions that must be learned, “on the spot,” with little to no training. It also fails to explain domain-specific imitation difficulties in highly trained, enculturated apes.

The case of enculturated apes reviewed here challenges the hypothesis that enculturation fundamentally alters one particular type of social cognition, imitation. Instead, these results indicate that the amount of training or imitation-specific experience an individual receives does not fundamentally or qualitatively change species difference in imitation performance. That is, experience, including the training of general processes such as executive functions, social learning-specific skills such as *do-as-I-do* or both, generally, improves great apes’ ability to reproduce transitive actions that are meaningful to them or that they have executed in the past and can recall from memory. However, such experiences seem to have little or no effect on apes’ ability to copy intransitive gestures or tool-use.

### 4.2. Motivational Accounts for Discontinuities

Yet, another possibility is that reported differences in the imitation performance of humans and apes (enculturated or non-enculturated) have less to do with the presence/absence of specialized mechanisms for imitation and social learning and more to do with differences in motivation. Perhaps, it might be useful to start with a textbook definition of motivation. According to Nevid [141] (p. 278), “The term motivation refers to factors that activate, direct, and sustain goal-directed behavior...Motives are the ‘whys’ of behavior—the needs or wants that drive behavior and explain what we do”. Given this definition, we might ask, what “activates, directs and sustains goal-directed behavior”? Cognitive mechanisms, of course! That is, cognitive processes dedicated to the representation of particular inputs and/or generation of specific outputs. We can also say that those very mechanisms are what “drives behaviors and explains what we do.” Cognitive mechanisms may be regarded as adaptations whose functions may be domain-specific (e.g., face detection, predator avoidance, mate identification) or domain-general (e.g., associative learning, causal reasoning, attention). Some complex cognitive mechanisms—such as theory of mind—involve both domain-general cognitive processes such as working memory and inhibition and specialized systems mediating mental-state representations [142]. They are considered adaptations in so far as they are believed to have been shaped by natural selection and maximize the fitness of the organism possessing said cognitive mechanism in a given niche. For a contemporary discussion of cognitive mechanisms as adaptations see Barrett [143].

To my knowledge, no study has systematically evaluated how different “motivators” affect social and imitation learning in great apes, enculturated or otherwise, despite the fact that prominent researchers have often alluded to significant differences in the “internal motivation” of humans’ and apes’ performance in social and imitation learning tasks [29]. Perhaps, such internal motivational differences may have to do with differences in how humans and chimpanzees attend to conspecifics versus other species. Specifically, Boesch [31] as well as de Waal and colleagues [144] have argued that apes are at a disadvantage in imitation studies where humans, rather than conspecifics, act as models. While virtually all of the studies summarized here, many of which report imitation (of different forms) among enculturated apes, used human models, there is some evidence consistent with the view that apes may attend to human models differently than conspecifics. Hattori and colleagues [145] using eye-tracking technology, have shown that humans and chimpanzees differ in how they modulate their attention in responses to specific social cues within and between species. In one study, humans and non-enculturated chimpanzees viewed images of conspecifics looking at an object (look), reaching toward an object (reach) or staring ahead (neutral). Consistent with prior work [146], results showed that chimpanzees and humans did not differ in their looking times when viewing faces in the neutral condition. However, while humans showed similar looking patterns regardless of whether the model was a chimpanzee or another human, chimpanzees looked significantly longer at the face and body of chimpanzee models than human models across most experimental conditions. Though, critically, chimpanzees also looked longer at the object (in both experimental conditions—look and reach) when a human was the model than when a chimpanzee was the model. Recall that Tomasello et al. [35] while reporting broad overlap in the imitation performance of 1.5 and 2.5 year-old human children and enculturated apes noted that enculturated apes produced more “end only” responses (copying the result of a given action without copying the particular means), than either child group. Such a result is expected, if subjects are fixating on objects, rather than the actions of the model.

These results provide some support for the notion that motivational differences, mediated by specific cognitive differences in visual attention, between humans and non-enculturated apes, may affect some forms of social learning in certain domains. However, it is unlikely that differences in visual attention alone can explain all domain-specific differences reported here (e.g., difficulties imitating intransitive actions relative to transitive actions on the body); Such a possibility is even more unlikely if this object-centric bias is absent in enculturated apes and their visual attention is more human like, as some have suggested [13,24]. Regardless, it is clear that motivational differences can and should be explained by specific cognitive differences, be they specific differences in executive functioning, attention or learning. Explaining results this way rather than by appealing to some amorphous or vague “motivational” difference, not only makes the problem more empirically tractable, it also makes an answer more likely.

### 4.3. Developmental Accounts for Discontinuities

Some have argued that the differences in the imitation skills of humans and apes may have to do with differences in cognitive development [32]. There are, in fact, significant differences in both the pace (i.e., when certain skills develop) and pattern (i.e., how development unfolds) of social cognitive development between humans and other apes [147]. For instance, while humans and apes share many gaze following skills [148], the developmental timing of these skills differs between species. In humans, simple forms of gaze following develops by six months with more complex forms of gaze following (e.g., around barriers and obstacles) appearing by 12 months [149]. In chimpanzees, the simplest forms of gaze following appear by 13 months [150], with more sophisticated forms of gaze following developing between 24 and 36 months [99,151], almost one to two years after humans.

There are also differences in the development of goal-directed actions. Human infants, by six months of age, attribute specific goals to others’ reaching actions [152]. By nine months, human infants frame others’ responses in terms of rational action goals [153,154]. Enculturated and non-enculturated apes, alike, develop similar skills after two to three years of age [99,155]. However, arguably, the most significant developmental discontinuity appears to be in joint attention [99,147,156]. While human infants, before 12 months of age, regularly engage others’ attention in triadic relationships, modulating their attention between an object and their caretaker, great apes fail to evidence the same behavior [93,147,156]. The same is true of enculturated apes [99,139]. Yet, these same great apes appear to understand and direct others’ behavior at later ages [155,157].

Might these developmental differences explain domain-specific differences in imitation performance? Joint attention may serve as a global or “general” mechanism directing observers’ interest to the subject of others’ attention. Such a skill is, presumably, critical for many (if not all) forms of imitation. In fact, there is evidence that in humans joint attention is related to imitation performance [158,159]. However, the evidence for a relationship between joint attention and imitation in enculturated apes is mixed with some showing a relationship [139] and others showing that apes, which fail to evidence any type of joint attention, nonetheless, succeed in certain social and imitation learning tasks [99]. Such discrepancies suggest that joint attention may be more relevant to the development of some forms of imitation than to others. One possibility is that a failure to engage in joint attention, while not essential for familiar imitation, may be essential for certain forms of novel imitation in particular content domains (e.g., transitive actions with objects that have salient end-states). In this case, the failure to develop joint attention may block the development of other cognitive skills or imitation-specific mechanisms. Future studies should, first, assess how basic social-cognitive processes including goal understanding, gaze following and joint attention, covary with performance on different familiar and novel imitation tasks across domains in enculturated and non-enculturated apes alike and second, how observed patterns of associations are alike and unlike those observed in younger and older human children as well as in adults.

Finally, some have questioned whether human and great ape comparisons are valid at all, given differences in rearing histories. According to these scholars, the rearing environment of all captive apes is impoverished relative to wild populations and, consequently, cognitive abilities are similarly impoverished [31,32,144]. Bard and Leavens [32], for instance, have even made direct comparisons between captive apes and infants in Eastern European orphanages. The conclusion of these authors is that results from most captive studies—if not all—are invalid because they lack external validity and are not generalizable [31,32]. While it is true that the rearing histories of captive apes differ from that of wild apes, there is no direct evidence that cognitive skills differ, fundamentally, between captive and wild populations. There is even less direct evidence for the view that the cognitive skills of captive great apes used in contemporary research are impoverished relative to wild populations. In fact, Kummer [160] (p. 35) has made the opposite point, noting that “…when we stop looking through ethology-tinted glasses we can see that ’unnatural’ [captive] environments may facilitate certain kinds of development considerably better than ‘natural’ ones”. In support of this claim, Tomasello and Call [34], in addition to summarizing the wide array of cognitive skills evidenced by captive apes, also point to cognitive skills such as tool-use (gorillas and bonobos), referential gesturing and symbolic (language-like) abilities (chimpanzees and bonobos) that have been reported in captive apes but are limited or absent, entirely, in wild populations. The present review similarly contradicts the view that captivity and rearing history has a negative effect on imitation performance. Instead, this review shows that while enculturation may enhance imitation performance, it does not appear to alter the overall pattern of apes’ successes and failures in particular task domains. This is a conclusion shared by various authors working in this and other cognitive domains [19,26,95].

### 4.4. Humans Possess Imitation-Specific Specializations

Why might humans have specialized social and imitation learning mechanisms? Lyons [161] and colleagues [162] and Gergely and Csibra [154,163,164,165] propose a plausible hypothesis regarding the ultimate cause of distinctively human imitation, specifically those involving novel transitive actions on objects, object–object actions and tool-use. According to Gergely and Csibra [154,163,164,165], the key to understanding this aspect of human imitation is by appreciating an important difference between human and non-human interactions with objects. Non-human object and tool-use appears to be entirely result-driven. That is, non-human tool users first identify consequences or end-states, such as extracting termites, and then look around for something that will help them achieve such results. Humans, by contrast, attend to both ends and means when learning from others [54,101,166] and faithfully copy both item-specific and motor-spatial-specific rules that have been associated with successfully imitating in various object-based tasks [55,84].

Another feature of great ape tool-use is that their function can be inferred from the raw material (see Tennie’s [4] “Zone of Latent Solutions”). As a result, this kind of tool-use is easily acquired by familiar imitation because the functional affordances of tools are understood from the start, priming semantic memory stores that contain that information. However, human tool-use is different in important respects. Much of it requires learning how to use tools whose functional affordances are unclear or unknown. As a contemporary example, consider a child learning how to use the television’s remote control. The functions of the various buttons are not obvious. Successfully learning how to use this tool requires attending to detailed manipulations without understanding their precise role in securing the ultimate result (i.e., finding a desired TV program).

As with tool-use, human dependence on language and manual gestures to communicate is likely to have placed unique pressure on our species, favoring those who possessed specialized mechanisms for rapidly and accurately imitating novel manual actions (i.e., intransitive gestures). Virtually all enculturated apes evidence significant difficulties initially learning and subsequently imitating intransitive gestures, regardless of familiarity [88,110,167,168] (cf., Table 1); a pattern starkly different from that seen in young human children [5,7,132]. A likely explanation for this discontinuity may have to do with the fact that in humans, gestures are intimately linked with another unique feature of our species, language. According to Goldin-Meadow [169], “…gesture forms a single system with speech and is an integral part of the communicative act”. In humans, gestures emerge early, at around 8 to 12 months [170]. These gestures are deictic, that is, they are used to point and refer to objects in the immediate environment. Human infants use these gestures, often, for the sole purpose of “sharing attention” with caretakers. In contrast, ape gestures are mostly imperative, associated with making a request, generally, a food request [130,171]. Researchers have pointed to the absence (or rarity) of deictic gesture in language-trained apes [110,172,173,174] to argue that this form of gestural communication is mediated by cognitive and conceptual mechanisms that appear to be absent in great apes. 

## 5. Future Directions

### 5.1. Characterizing Difficulties Copying Opaque Transitive Actions

Given the mixed results reported above suggesting that apes may have some difficulty copying certain opaque transitive actions, future studies should include a greater variety of opaque and transparent transitive actions directed toward the body and toward objects. Ideally, these actions should be matched so that the only difference is their opacity and transitivity. Variation in performance should be systematically analyzed in order to better characterize imitation within and across domains. This analysis should include an evaluation of individuals’ rearing histories and their familiarity with particular actions or objects (see Byrne and Tanner [63] for an excellent analysis of intransitive actions).

Another avenue of research is to identify covariances between opaque imitation and specialized bodily representational mechanisms as measured by mirror self-recognition (MSR) and imitation recognition (IR) [175,176,177]. Studying MSR and IR might be particularly useful because opaque imitation tasks are likely to tax bodily representations among other specialized representational systems necessary to resolve the correspondence problem [60]. Such studies should contrast covariances with these specialized systems as well as with various “general” processes [178] such as associative learning, attention, and WM. Do associations differ depending on the opacity and familiarity of the task? One might predict that abstract bodily representation measures (MSR, IR) should covary more with novel opaque imitation than with familiar opaque imitation. However, regardless of task familiarity, covariances between MSR and IR should be greater for opaque than for transparent imitation tasks. Conversely, general processes are more likely to covary with familiar opaque imitation tasks than with novel opaque imitation tasks.

### 5.2. Characterizing Difficulties Copying Intransitive Actions

Because intransitive gestures have been linked with language and communication development [169,179], one avenue of research might be to compare the imitation of intransitive gestures by language- versus non-language trained apes. Apes trained on visual languages such as ASL might be a particularly interesting test case because they have both linguistic and imitation-relevant motor training (i.e., learning manual signs). If language experience is key to learning novel intransitive gestures, then apes with language training should outperform those without language training, regardless of the type of language training (e.g., Lexigram vs. ASL). Alternatively, if motor-learning and imitation-relevant training is essential [109], then apes who received manual language training should out-perform those who received non-manual language training (e.g., lexigram). In humans, there is some evidence to suggest that, in fact, knowing a visual language like ASL, improves imitation performance, overall [180]. It is an open question whether the same is true of apes.

### 5.3. Characterizing Difficulties Copying Novel Actions with Objects 

As with characterizing intransitive gestures, in order to characterize species differences in the copying of novel actions with objects, it is critical to test individuals on a variety of object- and tool-based tasks. Comparing imitation performance across tasks within domains is critical as work with children has shown imitation performance on one task does not always predict imitation performance on other (superficially similar) tasks [84]. Characterizing associations and dissociations between tasks is key because there are, presently, no “standard” tasks, procedures, or measures in the social learning sciences. Consequently, little is known of the underlying cognitive features of commonly used tasks (e.g., Artificial Fruit Task [47] versus Doorian Fruit Task [104] versus Trap Tube Task [181]), including their association with other psychological processes. This is unusual. Most areas within the cognitive sciences have “gold-standard” tests. While these tasks have limitations, there is, nonetheless, general agreement about the underlying constructs that such tasks measure: the Flanker [182] and Stroop [183] Task in attention and executive control research, the Digit Span Task in WM research [184], the Sally-Anne and Smarties Task in Theory of Mind research [185], to name a few. The lack of a similar set of gold standard tasks (along with standard methods, measures and constructs) for imitation has significantly limited our ability to understand why apes and humans differ when imitating novel object–object and tool-based actions.

Future studies should take a feature- and content-based approach to better characterize species differences in the imitation of object- and tool-based tasks (see [84]). One useful framework is Thorndike and Woodworth’s [186] “Theory of Identical Elements.” That theory proposes that transfer learning between tasks is dependent on the number of features or “elements” those tasks share. Here, I have identified a number of elements that may distinguish human and ape imitation: task opacity, transitivity and familiarity. For transitive object- and tool-based tasks, the critical elements are likely to be familiarity (of the context and the objects) and whether the actions involve primary or tertiary relationships (egocentric: hand-object versus allocentric: object–object actions). Subiaul and colleagues [84] have suggested that another critical element is whether tasks involve encoding and copying item-specific rules (i.e., identifying and seriating discrete items), motor-spatial-specific responses (i.e., allocentric relationships), or both. Constructional praxis [140,187]—the ability to combine, assemble and join different pieces into a whole—might be another critical conceptual and cognitive skill underlying species differences in object- and tool-based imitation. Poti and Parenti [140] offer a useful framework by which to systematically study constructional praxis, focusing on both the products and the processes involved in this type of spatial reasoning. One important question for future research is whether individual differences (within and between-species) in the various components of constructional praxis predict object–object or tool-based imitation. A related question is whether species differences in constructional praxis may be explained by differences in more “general” cognitive processes, by specialized cognitive systems or the interaction/coordination of domain-general and domain-specific systems.

### 5.4. Data Sharing and Openness 

The field of comparative psychology is a notoriously closed field. This lack of openness and transparency has made independent analyses and replication difficult, if not, impossible. For this reason, comparative researchers should consider participating in Databrary [188], a federally-funded, online data repository that allows investigators to store and share raw data, including videos, of their research. The goal is to make science more open and data more widely available. Given the poor accessibility to non-human primate populations, especially enculturated ape populations, open access to research videos with these subjects would be a boon for the field. Such access—in addition to contributing to openness and transparency and improve the legitimacy of the field—would allow scientists to code for different responses, answer additional questions that were not initially explored and perform critical meta-analyses across studies. In short, there is little to lose but much to gain if comparative scientists participate in earnest in data sharing services such as Databrary.

## 6. Conclusions

While it is important to emphasize the continuities that exist between human and great ape imitation performance, it is critical that we not ignore the discontinuities that are also evident. Specifically, humans and other animals are likely to share imitation mechanisms that accurately match observed responses with familiar action representations stored in semantic memory. The likely mechanisms supporting the imitation of familiar responses across domains and tasks are probably domain-general processes including associative learning [36,52]. General processes [166] constrained by domain-specific social (communicative) inferences [189] may also explain continuities between humans and apes in tasks measuring rational imitation or goal emulation, where enculturated chimpanzees (and, perhaps, non-enculturated orangutans) appear capable of inferring and subsequently copying tool-use rationally. However, humans also appear to possess imitation skills that are either diminished or absent, altogether, in other apes. Broadly, apes appear to have difficulty copying novel responses involving transitive actions. However, apes also have difficulties copying, opaque actions and intransitive gestures, regardless of their familiarity. Because apes and human preschoolers share many of the same sensory and motor-skills (and, in the case of at least some enculturated apes, similar rearing environments), it is hypothesized that specialized mechanisms—rather than “general” processes—are most likely to mediate these species differences. Candidate mechanisms may include cognitive processes representing allocentric space [140] as well as specialized mechanisms for copying abstract motor-spatial rules [84], among other cognitive mechanisms such as motor simulation [190] and abstract representations of the body.

If, in fact, the cognitive structure of imitation is mosaic as my colleagues and I have suggested [40,46,57]—including domain-specific and domain-general processes depending on task and content domain—then we must use a more nuanced experimental approach in the comparative study of social and imitation learning. This approach must include a variety of tasks that assess different types of imitation learning and whose underlying component processes are well characterized. Imitation learning measures should prioritize first trial accuracy in experimental and baseline conditions, as any subsequent response inherently confounds vicarious (social) learning with individual (operant) learning. Finally, given that children and apes appear to posses various social learning skills in common, tasks, experimental conditions and statistical analyses must carefully control and consider the possibility that participants are applying one or more of these social and/or imitation learning mechanisms to solve a given problem. This is a particular concern because, depending on the task, individuals may come to the same solution using different cognitive processes. Such differences may represent individual differences within species (e.g., young human children may be more prone to emulation than older children) as well as between species (e.g., chimpanzees may be more prone to copying end-states—emulation—whereas human children may be more prone to copying specific means—action imitation). Given these considerations, the work reported here and elsewhere suggests that while the mechanisms that support the imitation of over-rehearsed and familiar responses are likely to be the same across a variety of species, the mechanisms that are involved in vicariously learning and copying responses with tools as well as intransitive gestures evidence both domain-specificity and phylogenetic distinctiveness. The specialization of social and imitation learning in humans may explain why humans are over-imitators, copying responses across different tasks and domains with high-fidelity, and possess cultural traditions that evolve within and across generations.

## Figures and Tables

**Table 1:** Summary of Great Apes’ Imitation Performance on Intransitive and Bi-Manual Tasks.

		Transparent-Visible Actions	Opaque-Non-Visible Actions	Transparent/Opaque
		Novel/Familiar		Novel
Study [Subject]	Species	Intransitive	Intransitive	Bi-Manual
*Viki* [22]	*Chimpanzee*	^	^	^
*Washoe* [88]	*Chimpanzee*	–	–	
*Nim* [89]	*Chimpanzee*	–	–	
*Kanzi* [53]	*Bonobo*			–
*Panbanisha* [53]	*Bonobo*			–
*Panzee* [53]	*Chimpanzee*			–
*KA* [18]	*Chimpanzee*	^	^	^
*SC* [18]	*Chimpanzee*	^	–	^
*Chantek* [90]	*Orangutan*			
*PRI* [91]*	*Chimpanzee*			
*Chantek* [92]	*Orangutan*	^	^	^
*PRI Chimpanzees* Alex [93]	*Chimpanzee*			
*Annet* [93]	*Chimpanzee*			
*Alexandra* [93]	*Chimpanzee*			
*Zura* [63]	*Gorilla*	^	^	
*Lili* [94]	*Chimpanzee*	+	+	

Note: Overall accuracy or percent imitated: 0–35 = – (poor)/ 36–60 = ^ (mixed results)/ > 60 = + (good to excellent). About coding: When individual response data was reported, only subjects’ first responses were analyzed. A “no response” was coded as a failure to imitate. * PRI = Primate Research Institute, Kyoto University. Did not provide data for individuals.

**Table 2:** Summary of Great Apes’ Imitation Performance on Novel Transitive Tasks.

		Transparent-Visible Actions		Opaque-Non-Visible Actions	
		Novel	Novel	Novel	Novel
Study [Subject]	Species	Object-Object	Transitive	Object-Object	Transitive
*Viki* [22]	*Chimpanzee*	–	^		
*Washoe* [88]	*Chimpanzee*				
*Nim* [89]	*Chimpanzee*				
*Kanzi* [53]	*Bonobo*	^	^		^
*Panbanisha* [53]	*Bonobo*	^	^		+
*Panzee* [53]	*Chimpanzee*	^	^		^
*KA* [18]	*Chimpanzee*		^		^
*SC* [18]	*Chimpanzee*		^		^
*Chantek* [90]	*Orangutan*		–		
*PRI* [91]*	*Chimpanzee*	^	–		–
*Chantek* [92]	*Orangutan*				
*PRI Chimpanzees* Alex [93]	*Chimpanzee*	–	–		
*Annet* [93]	*Chimpanzee*	–	+		
*Alexandra* [93]	*Chimpanzee*	+	^		
*Zura* [63]	*Gorilla*				
*Lili* [94]	*Chimpanzee*	–	^	*–*	^

Note: Overall accuracy or percent imitated: 0–35 = – (poor)/ 36–60 = ^ (mixed results)/ > 60 = + (good to excellent). About coding: When individual response data was reported, only subjects’ first responses were analyzed. A “no response” was coded as a failure to imitate. * PRI = Primate Research Institute, Kyoto University. Did not provide data for individuals.
